# Supplementary material for: Deficiencies of effectiveness of intervention studies in veterinary medicine: a cross-sectional survey of ten leading veterinary and medical journals
Source: PeerJ. 2016 Jan 28;4:e1649. doi: 10.7717/peerj.1649 (PMC4734056; doi:10.7717/peerj.1649)
Supplement: Supplemental Information 1 [file peerj-04-1649-s001.pdf]

Supplementary Note 1

Details on pilot study, sample size calculation and eligibility criteria for journal inclusion

Sample size

A pilot study on a different dataset was performed to assess the prevalence of RCTs over the total number of articles describing effectiveness of interventions in veterinary medicine and general medicine. All articles that were published in the first 6 months of 2006 in one veterinary journal (JAVMA) and one medical journal (JAMA) were assessed for RCT prevalence. A prevalence of 69,1% (29 RCTs/42 intervention articles) and 29,7% (8 RCTs/27 intervention articles) was identified for JAMA and JAVMA respectively. Using a formula for two proportions and equal group size [1] a minimal sample of 45 articles per group was required to have 90% power to detect a difference at a statistical significance of 1%.

We estimated how many journals we had to search and for which time span based on pertinent research data from a study by Giuffrida et al. [2]. This study identified 47 RCTs during a time period of 5 years in 2 of the veterinary journals included in the present survey, i.e., a mean of 4.7 RCTs per-journal per-year. Based on our pilot study we expected to find roughly one RCT per 3 EoI articles and that each journal should have published approximately 15 EoI articles per-year. Therefore, hand-searching 3 journals for each specialty (i.e., veterinary medicine and general medicine) for one year would be sufficient to obtain the required sample size. Considering a worst-case scenario, we included 5 journals per-discipline (i.e., a total of 10 journals).

Criteria for journal inclusion

To be eligible for inclusion in the study the journals must be in English, must have a broad scope (i.e., general and internal medicine) and must have been relevant in the field for a certain period.

The "VETERINARY SCIENCES" category of the 2013 ISI Journal Citation Report was sorted by decreasing impact factor. All the journals that focused on sub-specialties (e.g., Veterinary Microbiology, Veterinary Parasitology, etc.) or in non-English language were excluded. To endorse historical journals, all the journals that in the year 2000 were not published or had an impact factor lower than 1.0 were excluded. Aims and scopes of the remaining journals were evaluated on their websites until the first 5 journals presenting broad scope were identified: '*Veterinary Journal*'; '*Veterinary Record*'; '*Journal of Veterinary Internal Medicine*'; '*Journal of the American Veterinary Medical Association*'; '*American Journal of Veterinary Research*'.

In a similar fashion, 5 leading general medical journals were included. The category "MEDICINE, GENERAL AND INTERNAL" of the 2013 ISI Journal Citation Report was sorted by impact factor. All the journals that focused on a sub-specialty were excluded. The scope of the remaining journals was evaluated on their websites until 5 journals presenting broad scope published at least since 2000 were identified. The 5 medical journals included in the study were: '*New England Journal of Medicine*'; '*the Lancet*'; '*Journal of the American Medical Association*'; '*British Medical Journal*'; '*Annals of Internal Medicine'* (Table 1).

Supplementary Note 2

Additional details on data extraction

The following data items were recorded during the data extraction procedures.

Number of full original articles

The total number of full original articles was recorded because in previous studies in other specialties [3,4] the prevalence of RCTs was provided as: RCTs/All published articles.

Reports eligible as full original articles: primary research, including subgroup analysis or follow-up of previous articles; case series, defined as original reports including more than one patient.

Reports not eligible as full original articles: Single case reports; Secondary research, including systematic reviews and non-systematic reviews; Qualitative studies, letters, point of view, commentaries, clinical queries, i.e., all articles without the original research format (i.e., Introduction-Materials and Methods-Results-Discussion); Mathematical modelling of pre-published database.

Number of articles evaluating effectiveness of interventions (EoI)

"*Effectiveness*" was defined as "*evaluation of benefits*" of an intervention. "*Interventions*" were defined as "*act used to improve health, to treat a particular condition or disease in process or to prevent development of a particular condition or disease*" [5,6]. For the purpose of this review, legislation changes and taxes were not considered interventions. Exclusively *in vitro* studies were not included in this category.

Reports eligible as EoI articles: Case series, case-control studies, cohort studies, analytical cross-sectional studies, non-randomised controlled trials, and RCTs were included in this category if evaluated the desired effect of an intervention *in vivo*.

Reports not eligible as EoI articles: (1) reports focusing solely on undesired and adverse effects of the intervention, e.g., studies of hemodynamic changes after administration of an anaesthetic intervention; (2) reports focusing on an outcome that is the measurement of the intervention itself, e.g., pharmacokinetic and pharmacodynamics studies, studies of hormonal stimulation for diagnostic tests. Instead, studies evaluating changes in outcome that may have a direct clinical significance (e.g., increase in vitamin D levels after exposure to UVB light; decrease in white blood cells after an antimicrobial treatment, etc.) were considered EoI articles; (3) reports of accuracy of a diagnostic technique (i.e., sensitivity and specificity or mean difference). Instead, articles evaluating the effect of a diagnostic technique on clinically important outcomes for the patient were considered EoI articles.

Number of EoI articles that described surgical interventions

The type of intervention was categorised in surgical/non-surgical, as researches of surgical interventions face different challenges regarding several aspects, including study design [7]. EoI articles were considered “surgical” when (1) the intervention required cutting of the skin. Needle-related procedures (e.g., amniocentesis, etc.) were not considered surgical procedures; (2) the difference between the control and the experimental group was in the presence, type or technique of the surgical procedure. If the difference between the control and the experimental

group was a medication given after/before a surgical procedure the trial was not considered surgical.

Number of RCTs

Studies were defined RCTs based on the US National Library of Medicine 2008 definitions for the Publication Type terms 'Randomised Controlled Trial' and based on the definition of the Cochrane glossary [8]. All the reports with allocation to interventions described as randomised were included [9]. A study was classified as "a RCT" when (1) at least two interventions were compared; (2) and randomisation was mentioned.

Studies based on RCTs that are not the primary outcomes of RCTs, subgroup analyses or long-term outcome of previously published RCTs were also considered RCTs if randomisation was maintained. Articles were included when they reported on "randomly" allocated interventions, even when their actual allocation was "nonrandom" (e.g., alternation, date of admission, etc.). Crossover studies (including Latin square design studies) were considered RCTs if the patients were randomly assigned to the treatment groups. If the word "random", "randomly", "randomised" or "randomised" was not used to describe allocation of the patients to the treatment, the study was not considered a RCT.

Number of RCTs that included real patients

We evaluated if RCTs involved real clinical patients or non-patients, i.e., voluntary individuals or experimental animals. Real clinical patients were defined as "*the population that presents the condition that needs to be treated or prevented and that will benefit of the intervention once established*". Articles were considered to

include real clinical patients when these individuals or animals: (1) suffered from a spontaneous disease; and (2) were exposed to real-life conditions. Only animals kept in their usual environment and owned by their usual personnel were eligible for inclusion in this category. For example pet animals owned by private individuals and farm animals owned by farmers are considered real clinical patients. Shelter animals are considered real clinical patients only in case an intervention is specifically directed to treat a condition that occurs in shelters.

Non-patients refer to: (1) Animals suffering from induced diseases; (2) Animals maintained in laboratory conditions (except when the laboratory animal is the final beneficiary of the intervention); (3) Healthy individuals or animals, except when an intervention is specifically planned for healthy individuals or animals, e.g., the use of a particular diet, neutering of pets, etc.

Assessment of reporting of key methodological domains in RCTs

The following protocol was applied for the assessment of reporting key methodological domains in RCTs. Two operators (ND, LNPC) independently assessed the RCTs. In case of disagreement, an arbiter was consulted (RMR). Firstly, the *materials and methods* section was thoroughly read and relevant information was highlighted. Then, key words were searched using the search function of Portable Document Formats (PDF)s to find additional information that was not reported in the *materials and methods* section. The search words included: “power”, “sample” and “size” for power calculation; “primary”, “main”, “outcome” and “endpoint” for primary outcome; “random”, “allocat” for randomisation and allocation concealment; “blind”, “mask”, “aware”, “know”, “inform” for blinding domains; “95”, “CI” and “interval” for effect size estimation; “intent”, “analysis” and “attrition” for handling

of attrition. To avoid inappropriate exclusion of pertinent items, all grammatical derivatives of these search terms were applied during these searches. Finally, each methodological domain was scored “yes” if adequately reported, or “no” if not adequately reported.

Evaluation of additional data than the published article – To avoid inappropriate exclusion of eligible articles, full-texts of protocols, supplements, and previous or accompanying manuscripts, which were explicitly mentioned in the main text were also assessed. In the case that no references to supplementary material were present in the main text, only the published report was assessed.

Key methodological domains

We evaluated the following key methodological domains [10]:

Primary outcome- Authors explicitly reported a primary outcome in the published article. If a primary outcome was not explicitly described, we considered the outcomes reported in the sample size estimation. When a primary outcome was not explicitly specified in the article or sample size calculation the paper was classified as “not reporting a primary outcome”. We recorded whether the primary outcome was retrieved from the power calculation or from a proper sentence.

Power calculation- Authors reported a power calculation that was performed *a priori* to estimate the sample size. Power calculations performed after completion of the study were not considered.

Random sequence generation- Authors explicitly described the methods and the type of randomisation to generate the random list. Two features were required to be listed as “yes” [11]: (1) Explanation of the method by which the random sequence was generated (i.e., computer, coin tosses, etc). A statement that a statistician

performed the sequence generation was also valid. (2) Explanation of the type of randomisation, e.g., simple randomisation, permuted block (to avoid imbalances in allocation), stratification (to balance the distribution of certain baseline risk factors), or a combination of these techniques.

Allocation concealment- The methods used to prevent the individuals enrolling trial participants from knowing or predicting the allocation sequence in advance (i.e., the method of preventing study personnel from having awareness of treatment assignment before enrolling; [11]), were described in the article. Acceptable methods, among others, are: Central, Pharmacy, Opaque sealed envelopes, etc.

Blinding- Definitions such as single, double or triple-blinding were not considered sufficient to explain who was blinded and to what [2,12].

Blinding of participants- The article explicitly described that participants were unaware of participants' group allocation. Blinding of participants in medical articles referred to blinding of patients. Blinding of participants in veterinary articles referred to blinding the owners of the animals.

Blinding of personnel- The article explicitly describes that operators involved in the care of participants were unaware of participants' group of allocation.

Blinding of outcome assessors- The article explicitly describes that outcome assessors were unaware of participants' group of allocation.

Intention-to-treat- The article explicitly mentions that the analysis was made on an "intention-to-treat" basis.

Effect size estimation methods- Results (in particular for differences between groups) are provided with methods that estimate the effect size (i.e., risk ratio, odds ratio, mean difference, etc.) with confidence interval, rather than solely "p values".

For the purpose of the analyses, the domains "primary outcome", "power calculation", "random sequence generation", "allocation concealment", and "use of estimation methods" were considered always feasible, while the domains "blinding of participants", "blinding of personnel", "blinding of outcome assessors" and "intention-to-treat" were considered occasionally feasible, depending on study characteristics.

Additional data extracted for each article

The following information was extracted from each article: Volume, Issue, Title, Nationality of the affiliation of the first author, Objective or hypothesis of the study.

Additional data extracted for each RCT

The following information was extracted from each RCT:

Number of participants - The total number of participants of each RCT was extrapolated. If more than one RCT with the same treatments were described in the same article, the total number of patients randomised was used. If the RCTs had different treatments (i.e., one placebo-controlled, and one active-controlled) the number of patients randomised in the placebo controlled RCT was employed. If a RCT and a non-randomised study were described in the same article, only the number of patients randomised was used.

Main publication of the RCT- We purposely included both first publications relating to particular trials than secondary publications of trials (i.e., study reporting subgroup analysis, cost-effectiveness analysis, long-term outcomes), prompt that the randomisation was still active. For further analyses such RCTs were classified as "secondary publications", while articles reporting the first results of a trial were classified as "primary publications".

Associated with non-randomised material- RCTs were categorized as "standalone" or "supplemented" based upon the presence of additional non-randomised articles (i.e., in vitro or prospective data) reported in the same article of the RCT.

Self-defined as RCT- Initially, we purposely used a broad definition of RCT (i.e., trials in which interventions are randomly allocated) to avoid underestimation of the proportion of randomised trials due to poor terminology reporting (i.e., investigators randomly allocate interventions but ignore the terminology "randomised controlled trial"). By the other side, the lack of explicit delineation of a randomised trial as "RCT" can be associated to a lack of reconnaissance as "RCT" by the investigators, and therefore to poorer reporting and higher risk of certain bias. Therefore, we further classified the randomised trials to account for the explicitly self-recognisant RCT (called "manifest RCT" in this piece of work) and for the others, randomised trials that did not recognise themselves as RCT (called "unstated RCT" in this piece of work). All the randomised trials that were registered in a trial repository were automatically included in the "manifest RCT" category. Trials not registered, needed to report the typical design terminology "randomised" (or "randomised"), "controlled" (or "clinical"), "trial" (or "study") somewhere in the main article to be classified as a "manifest RCT". This terminology did not have to be reported in a

specific section of the article. The old definition of "randomised field trial" was also considered acceptable. Randomised trials that were not registered in a trial repository and did not use this terminology somewhere in the article were categorised as "unstated RCT". A sub-category based on type and self-reconnaissance as RCT is: "parallel manifest RCT", which is created to encompass all the randomised trial that have the more classical design (parallel) and that are aware of being RCT.

References

1. Kirkewood, B. R., Sterne, J. A. Essential medical statistics. Oxford: Blackwell Science Ltd; 2003.

2. Giuffrida, M. A., Agnello, K. A., & Brown, D. C. Blinding terminology used in reports of randomized controlled trials involving dogs and cats. *J Am Vet Med Assoc*. **241**, 1221-1226 (2012).

3. Sjögren, P., & Halling, A. Randomised controlled trials and publication trends in periodontal research during 1980-2000. *J Clin Periodontol*. **29**, 1112-7 (2002).

4. Momeni, A., Becker, A., Antes, G., Diener, M. K., Blümle, A., & Stark, G. B. Evidence-based plastic surgery: controlled trials in three plastic surgical journals (1990-2005). *Ann Plast Surg*. **61**, 221-225 (2008).

5. Farlex Medical Dictionary. Definition: Intervention. Available: http://medical-dictionary.thefreedictionary.com/intervention, accessed on June 5 2014.

6. Merriam-Webster Encyclopaedia Britannica. Definition: Intervention. Available: http://www.merriam-webster.com/medical/intervention, accessed on June 5 2014.

7. McCulloch, P. No surgical innovation without evaluation: the IDEAL recommendations. *Lancet*. **374,** 1105-1112. (2009).

8. Glossary of Cochrane terms. Available: http://community.cochrane.org/glossary, accessed on March 6 2015.

9. Schulz, K. F., Chalmers, I., Grimes, D. A., & Altman, D. G. Assessing the quality of randomization from reports of controlled trials published in obstetrics and gynecology journals. *JAMA*. **272**, 125-8 (1994).

10. Chan AW, Altman DG. Epidemiology and reporting of randomised trials published in PubMed journals. Lancet. 2005;365(9465):1159-62.

11. Moher, D., et al. CONSORT 2010 Explanation and Elaboration: Updated guidelines for reporting parallel group randomised trials. *J Clin Epidemiol*. **63**, e1-37 (2010).

12. Haahr MT, Hróbjartsson A. Who is blinded in randomized clinical trials? A study of 200 trials and a survey of authors. Clin Trials. 2006;3(4):360-5.

Supplementary Data

Other characteristics of RCTs evaluated

Less than a half (41.2%; 47/114) of the veterinary randomized trials were classified as "explicit RCTs", while all RCTs in general medicine were categorised as "explicit RCT" (100%; 60/60). The vast majority of the medical RCTs (85%; 51/60) were categorised as "parallel explicit RCTs" (i.e., parallel RCTs using the RCT terminology), while only one third of the veterinary RCTs (35.1%; 40/114) were categorised as such.

Some veterinary articles (10.5%; 12/114) had additional in vitro or other non-randomized evidence included in the same publication of the RCT. None of the medical RCTs presented accompanying non-randomized evidence in the same article.

One article in veterinary medicine (0.9%; 1/114) was identified as a secondary publication of a previously published RCT, while 13.3% of the medical articles (8/60) were secondary publications of trials.

Supplementary Table S1

Binary logistic regression outcome. Association between journal and prevalence of RCTs. In this analysis the ORs represent the odds of publishing EoI studies with a randomized controlled design, compared with JAVMA. Medical journals had from 6 to 15 times the odds of publishing a RCT compared with one of the veterinary journals.

			95% CI for ORs		
		ORs	Lower	Upper	P value
Indicator	JAVMA				
	Lancet	15.400	6.441	36.818	.000
	NEJM	15.060	6.777	33.466	.000
	JAMA	11.183	4.632	27.001	.000
	BMJ	7.537	2.981	19.057	.000
	AJVR	6.926	2.726	17.599	.000
	Annals	6.286	2.269	17.417	.000
	Vet J	1.897	.880	4.089	.103
	Vet Rec	1.833	.702	4.786	.216
	JVIM	1.659	.712	3.864	.241
